# Risk Factors for Failure of Calcaneal Lengthening Osteotomy in Children and Adolescents With Planovalgus Foot Deformity: A Retrospective Study

**DOI:** 10.7759/cureus.43157

**Published:** 2023-08-08

**Authors:** Joe Mehanna, Abir Massaad, Ayman Assi, Joe Rassi, Alexis Atallah, Ismat Ghanem

**Affiliations:** 1 Laboratory of Biomechanics and Medical Imaging, Saint Joseph University of Beirut, Beirut, LBN; 2 Orthopedic Surgery, Hôtel-Dieu de France Hospital, Beirut, LBN

**Keywords:** planovalgus deformity, idiopathic, cerebral palsy, children, calcaneal lengthening

## Abstract

Introduction: The calcaneal lengthening procedure (CLP) is a well-known surgical technique used for the correction of symptomatic planovalgus foot deformities. Literature shows a significant rate of relapse and undercorrection of the foot post-operatively. Factors determining the failure or success of CLP are still not well understood. The purpose of this retrospective study was to assess the most significant factors related to the failure of this procedure.

Methods: A case-control retrospective study was conducted on 50 patients (80 feet) aged 12.4±2.5 years who underwent CLP. A clinical (demographic parameters, etiology, Gross Motor Function Classification System (GMFCS) level) and radiological evaluation were assessed preoperatively and repeated postoperatively at 56.5±32.5 months. Two methods of osteotomy bone fixation were studied: K-wires vs. eight-plate. Standing anteroposterior (AP) and lateral (L) radiographs were done, and the following radiographic parameters were measured: calcaneocuboid (CC) joint subluxation classified into normal, moderate, and severe (L); AP and L talo-first metatarsal (T1MT) angle; AP talonavicular (TN) coverage angle; AP and L talocalcaneal (TC) angle; calcaneal pitch (CP) angle; and L talo-horizontal (TH) angle. Mosca’s criteria were used for clinical and radiological assessments. The association between demographic data, clinical and radiological results, and the variation between preoperative and postoperative angles were studied. The main risk factors affecting clinical results and CC joint subluxation were investigated (logistic regression and analysis of covariance (ANCOVA)).

Results: Satisfactory clinical results were associated with satisfactory radiological ones on Mosca’s criteria (p<0.001). The use of an eight-plate for osteotomy fixation gave better results than K-wires (79% vs. 59%). Radiological angles were improved in both techniques postoperatively (increase of CP and L-TC and decrease of AP-T1MT, AP-TC, AP-TN, and L-T1MT, all p<0.05). Non-satisfactory clinical results were associated with a high GMFCS level, a low preoperative AP-TN coverage angle, and a low preoperative CP angle (R^2^=0.45). Both a young age and a low CP angle preoperatively were associated with CC subluxation (R^2^=0.31).

Conclusion: The neurological status and the severity of the planovalgus foot deformity preoperatively were the main risk factors affecting clinical outcomes after CLP. However, young age and the severity of the deformity preoperatively were the main risk factors behind CC joint subluxation affecting CLP outcomes.

## Introduction

Flatfoot deformity is the most common cause of consultation in children and is attributed to joint and ligamental instability [1]. During the first years of life, approximately 97% of children have planovalgus foot deformity, and this rate decreases to 44% between three and six years to stabilize between 15% and 23% in adulthood [2]. Planovalgus foot deformity is mostly idiopathic and is less frequently related to neurological, dystrophic, or traumatic causes [3]. Studies showed that treatment is not recommended for an asymptomatic flatfoot deformity with no alteration in daily life activities, while a symptomatic foot with pain and instability requires intervention [4].

Analgesic drugs, physiotherapy, and orthosis are commonly prescribed for symptomatic flatfoot deformity with equivocal results [5]. Surgical treatments such as repositioning procedures (calcaneal-stop screw, subtalar implant method), osteotomies (medial and lateral columns lengthening), and triple arthrodesis are recommended for feet resistant to the aforementioned conservative treatment with progressive and persistent symptoms [6]. Among all surgical treatments, the calcaneal lengthening procedure (CLP) is widely used in patients with flexible and symptomatic planovalgus foot deformity [7]. Many techniques were adopted to minimize failure and undercorrections following CLP such as the ones developed by Evans et al. [8], which consist of taking a tibial bone graft and inserting it in the anterior distal part of the calcaneum. This technique was modified later by Mosca et al. [9]. However, many failures were reported when it came to surgical procedures where Steinmann pins or K-wires are used for the osteotomy fixation, such as subtalar joint damage, pain, under-corrections, and calcaneocuboid (CC) joint subluxation [10]. While it has been demonstrated that preoperative foot morphology seems to be related to surgical failure [3], the clinical and radiological factors as well as the technique used during surgery were not thoroughly investigated in the literature. Clinicians dealing with the surgical management of planovalgus foot deformity should be aware of the most significant factors that could influence outcomes.

The primary objective of this paper is to identify the main predictors of failure of CLP. The secondary objectives are to evaluate the factors behind the occurrence of CC joint subluxation. We hypothesized that many clinical and radiological factors and the addition of some tips during surgery, as well as the use of a more stable internal fixation of the osteotomy site, may contribute to a better outcome.

## Materials and methods

Patients

This is a retrospective study that was approved by the ethical committee of our institution, Saint Joseph University of Beirut (approval no. CEHDF, tfem/2019/60). A consent form was signed by all the participants or their legal guardians. The indications for surgery were pain in the idiopathic planovalgus foot and pain and/or asymmetric plantar overload in the non-idiopathic planovalgus foot that was expressed by skin redness or ulceration, progressive hallux valgus deformity, or interference with shoe or ankle foot orthosis (AFO) wear.

A total of 62 (100 feet) consecutively operated patients between 2011 and 2018 were selected from the registry of a single surgeon operating at the university hospital. Patients less than 18 years of age at the time of CLP, having a planovalgus foot deformity, and with a postoperative follow-up of more than two years were included. In order to reduce the confusion bias due to retrospective studies, those who underwent additional medial column surgeries, distal advancement of the tibialis posterior tendon, plication of the talonavicular capsule, osteotomies, had a rigid flatfoot or had incomplete clinical or radiological records were excluded. Out of 62 patients, a total of 50 (80 feet) with an average age of 12 years, aged between 7 and 18 years, and meeting the inclusion criteria were reviewed for this study.

Surgical technique

**Figure 1:**
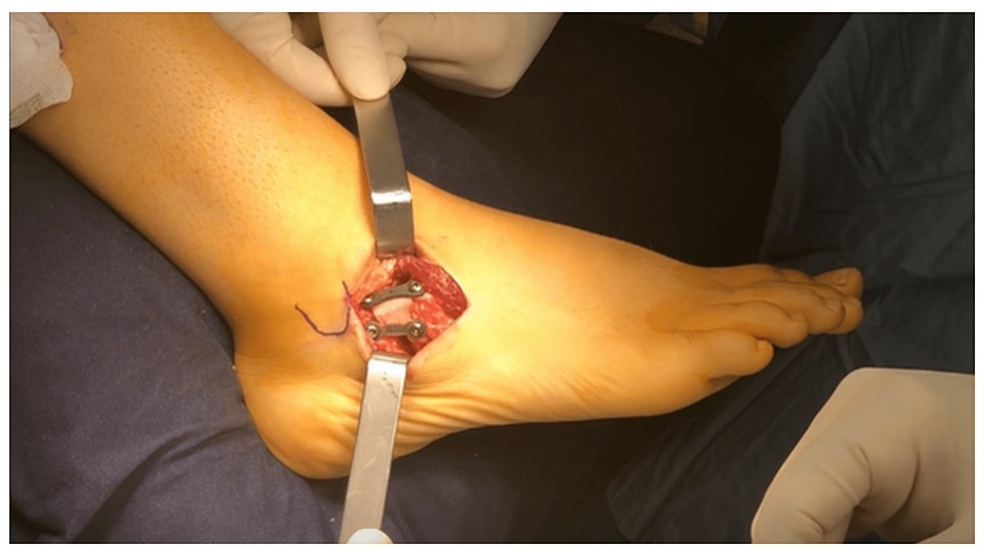
Osteotomy fixation with two eight-plates, one dorsally and the other laterally

A few modifications were introduced to the original surgical technique previously described by Evans et al. [8]. First, the bone graft was harvested from the middle third of the fibula and not from the ileal crest in an effort to decrease postoperative pain. The fibular graft was trapezoidal in shape (1 cm to 1.5 cm depending on the patient’s age and size) and was harvested from the middle third of the fibular shaft at least 10 cm above the lateral malleolus to avoid iatrogenic ankle valgus. The graft was inserted at the dorsolateral aspect of the open wedge osteotomy site to correct both the midtarsal and forefoot valgus as well as the foot planus deformity. Second, in the early cases of this cohort, CLP was performed without longitudinal division of the plantar fascia. In the remaining cases, the plantar fascia was divided longitudinally down to the level of the abductor digiti-minimi to facilitate 3D calcaneal displacement following the procedure. This was called extensive lateral release (LR) as compared to the limited release performed in early cases. The osteotomy site was fixed using K-wires for early cases and two eight-plates in later cases. The first plate was put dorsally, and its curvature was increased to push the distal fragment plantarily, thereby trying to avoid dorsal subluxation. A second plate was added laterally to increase stability (Figure 1).

**Figure 2:**
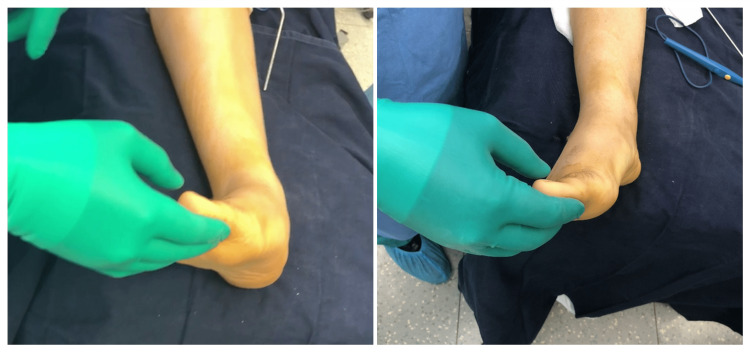
Immediate correction of the valgus foot deformity postoperatively

Achilles or gastrocnemius lengthening was added in some cases following CLP. The latter usually unmasks the associated short triceps. Postoperatively, a clear correction of the foot deformity is noticed (Figure 2).

A short, non-weight-bearing cast was applied postoperatively for four to five weeks, followed by a weight-bearing cast for two to three weeks. Physical therapy with passive and active motion of the foot and ankle was started after cast removal and continued for three to six months.

Methods of assessment

Patients were assessed clinically and radiologically by two authors of this study per Mosca’s criteria [11]. Clinical results were considered satisfactory if all six of the following conditions were satisfied: 1) the valgus deformity of the hindfoot has been corrected; 2) the longitudinal arch has been created; 3) the medial prominence of the talar head has been eliminated; 4) pain and callus under the head of the talus have been eradicated; 5) ulceration has not recurred; and 6) the tolerance to a brace or shoes has improved. Otherwise, the clinical result was considered unsatisfactory.

**Figure 3:**
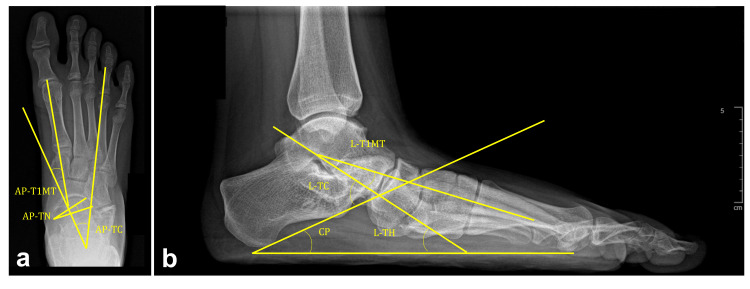
Anteroposterior and lateral weight-bearing radiographs a: Radiography revealing the AP-T1MT, AP-TC, and AP-TN coverage angle; b: Radiography showing the L-T1MT, L-TC, L-TH, and CP angle AP-T1MT: Anteroposterior talo-first metatarsal, AP-TC: Antero-posterior talocalcaneal, AP-TN: Anteroposterior talonavicular, L-T1MT: Lateral talo-first metatarsal, L-TC: Lateral talocalcaneal, L-TH: Lateral talohorizontal, CP: Calcaneal pitch

Anteroposterior (AP) and lateral (L) weight-bearing radiographs were performed preoperatively and at the last follow-up following surgery, at 56.5 months on average (minimum to maximum: 24 to 89 months). The talonavicular (TN) coverage angle was measured on the AP view (Figure 3a). The calcaneal pitch (CP) angle, talohorizontal (L-TH) angle, and CC joint subluxation were measured on the L view. The talo-first metatarsal (T1MT) and talocalcaneal (TC) angles were measured on both AP and L views (Figure 3b).

All pre and postoperative measurements were accomplished by an orthopedic resident using the Picture Archiving and Communication System (PACS) software (Centricity, GE Healthcare, Chicago, Illinois, USA). Radiological results were considered satisfactory if at least two of three angles were within normal limits according to Mosca’s criteria: the L-T1MT angle ranges between -7^o^ and 20^o^, the L-TH angle between 15^o^ and 37^o^, and the CP angle between 15^o^ and 30^o^.

Walking children and adolescents with or without assistive devices (walkers, crutches) were classified according to the Gross Motor Function Classification System (GMFCS) levels (I/II or III/IV) [12] and planovalgus etiology (idiopathic vs. non-idiopathic). Non-idiopathic feet were stratified into two groups: neurologic (cerebral palsy) or dystrophic (connective tissue or congenital diseases), and surgical technique (eight-plate vs. K-wire, extensive vs. limited LR). Patients with non-neurological planovalgus foot deformity were conventionally classified into the group GMFCS I. All the patients classified as GMFCS I have pain during school activities or an intolerance to shoes or leg braces. Foot morphology was never considered a selection criterion for surgery. 

**Figure 4:**
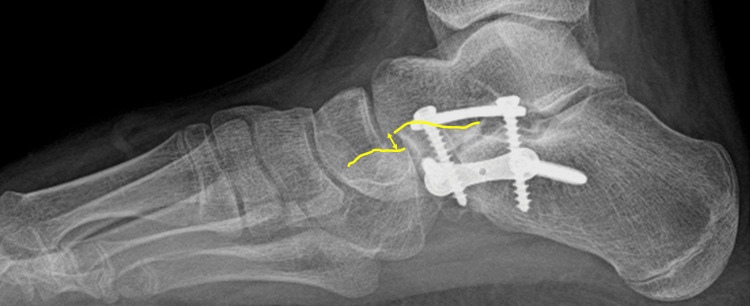
Measurement of CC joint subluxation on the lateral weight-bearing radiograph The CC joint subluxation is defined by the discontinuity between the upper dorsal calcaneal and the cuboid aspects and is measured by the distance between both of them (double arrow). CC: Calcaneocuboid

The CC joint subluxation was measured on the lateral weight-bearing radiographs and classified into normal (<2 mm), moderate (between 2 mm and 4 mm), and severe (>4 mm) (Figure 4). Calcaneocuboid joint pain was frequently present in severe forms. In five cases, proper assessment of the CC joint subluxation was impossible because of poor radiological quality.

Statistical analysis

The association between age, gender, etiology, ambulatory status (GMFCS score), surgical technique, and treatment outcomes (clinical and radiological) was evaluated using the chi-square test. Preoperative and postoperative radiological measurements were compared using the Wilcoxon test. To investigate the major determinants of the clinical result, logistic regression was computed while taking into account as independent variables the factors that were significant in the univariate analysis. To probe the main risk factors contributing to a CC joint subluxation postoperatively, a linear regression (analysis of covariance (ANCOVA)) was computed.

## Results

Descriptive and univariate analysis

A total of 50 patients with 80 planovalgus feet were included in the study (Table 1).

**Table 1:** Demographic data

Variables	Values
Patients (n)	50
Age (mean ± SD) (min-max)	12±3 (range 7 to 18) years
Follow-up (mean ± SD) (min-max)	56.5±32.5 (range 24 to 89) months
Gender	53 M
	27 F
Operated side (n)	8 left
	12 right
	30 Both
Operated feet (n)	80

**Figure 5:**
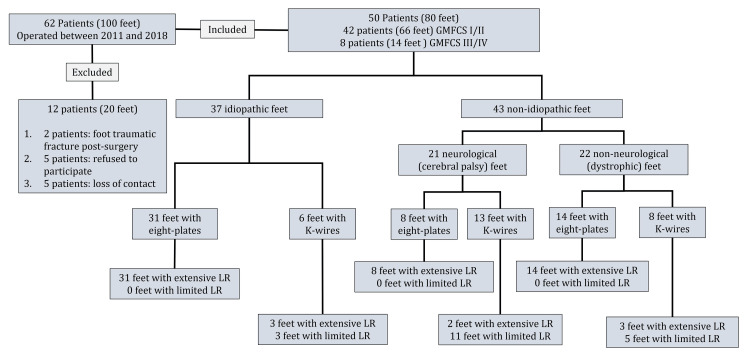
Study samples and subgroups GMFCS: Growth Motor Function Classification System, LR: Lateral release

Age at the time of surgery ranged from seven to 18 years. Forty-two patients (66 feet) were independent walkers (GMFCS levels I/II), and eight patients (14 feet) needed assistance while walking (GMFCS levels III/IV). Thirty-seven feet were classified as idiopathic and 43 as non-idiopathic (21 neurological (cerebral palsy) and 22 non-neurological (dystrophic). Concerning the surgical technique, K-wires were used for 27 feet and eight-plates for 53 feet. Limited LR was performed in the first 19 feet and extensive LR in the remaining 61 feet (Figure 5).

According to Mosca’s criteria, the results were classified as satisfactory in 82% of feet (n=54/66) with GMFCS levels I/II, compared to only 29% (n=4/14) in children with GMFCS III/IV. The rate of clinically satisfactory results was 38% (n=8/21) in neurologic foot deformity (cerebral palsy) and 85% (n=50/59) in non-neurologic (idiopathic or dystrophic) patients. The satisfaction rate was 79% (n=42/53) when eight-plates were used in osteotomy fixation and 59% (n=16/27) in the K-wires technique. The satisfaction rate according to Mosca’s criteria was 82% (n=51/62) in feet that did not show any CC joint subluxation, 50% (n=4/8) in feet with moderate subluxation, and 40% (n=2/5) in feet with severe subluxation. Clinical results were significantly associated with radiological results (according to Mosca), GMFCS level, etiology (idiopathic/non-idiopathic, cerebral palsy/dystrophic), CC subluxation, and the nature of the LR (limited vs. extensive) (Table 2).

**Table 2:** Associations with clinical results and satisfaction rate according to Mosca’s criteria GMFCS: Growth Motor Function Classification System, CC: Calcaneocuboid, LR: Lateral release

Variables	Satisfactory clinical result (no. of feet)	Non-satisfactory clinical result (no. of feet)	Clinical satisfaction rate (%)	p-value
Age <10 years	11	5	69	0.928
Age between 11 and 15 years	40	15	73	
Age >16 years	7	2	78	
Male	40	13	75	0.436
Female	18	9	67	
Satisfactory radiological result	58	16	78	<0.001
Non-satisfactory radiological result	0	6	0	
GMFCS levels I/II	54	12	82	<0.001
GMFCS levels III/IV	4	10	29	
Idiopathic flat foot	33	4	89	0.002
Non-Idiopathic flat foot	25	18	58	
Cerebral palsy	8	13	38	0.013
Dystrophic	17	5	77	
Normal CC joint	51	11	82	0.019
Moderate CC subluxation	4	4	50	
Severe CC subluxation	2	3	40	
Eight-plates	42	11	79	0.058
K-wires	16	11	59	
Limited LR	9	10	47	0.005
Extensive LR	49	12	80	

Comparison of radiographic angles (preoperative vs. postoperative)

**Figure 6:**
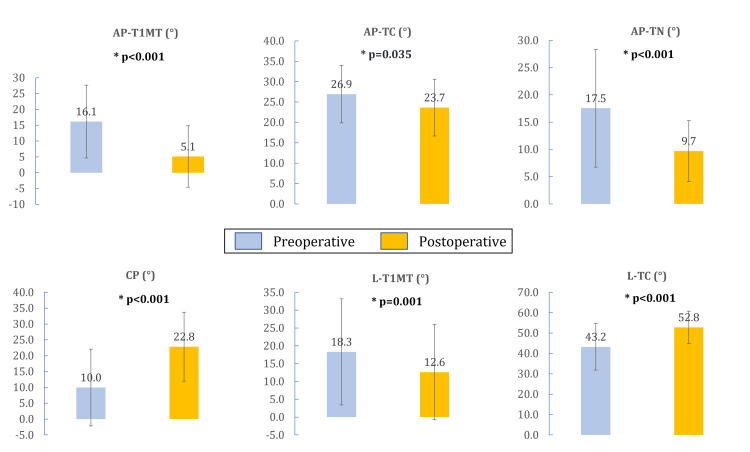
Variation of angles with the calcaneal lengthening procedure L-T1MT: Lateral talo-first metatarsal angle, L-TC: Lateral talocalcaneal angle, CP: Calcaneal pitch angle, AP-T1MT: Anteroposterior talo-first metatarsal angle, AP-TC: Anteroposterior talocalcaneal angle, AP-TN: Anteroposterior talonavicular coverage angle

The AP-T1MT, AP-TC, AP-TN (increasing coverage), and L-T1MT angles significantly decreased postoperatively, while CP and L-TC increased (all p<0.05) (Figure 6). The L-TH angle improved from 33±12^o^ preoperatively to 29±9^o^ but this was not statically significant (p=0.067).

Multivariate analysis

The logistic regression was computed to evaluate determinants of the clinical result variable included as dependent variable: preoperative AP-T1MT angle, preoperative AP-TC angle, preoperative AP-TN angle, preoperative CP angle, preoperative L-T1MT angle, preoperative L-TC angle, GMFCS I/II, GMFCS III/IV, planovalgus etiology (Idiopathic, dystrophic, cerebral palsy), surgical technique (extensive LR, limited LR). These independent variables were chosen since they were shown to be significant in the univariate analysis. 

A non-satisfactory clinical result (R^2^=0.45) was associated with a high GMFCS level, a decreased preoperative AP-TN coverage (increased angle), and a decreased preoperative CP angle (standard coefficients: 0.42, 0.21, -3.17 respectively; logistic regression).

Moreover, postoperative CC subluxation was associated (adjusted R^2^ = 0.31) with a low preoperative CP angle and a young age at surgery (standardized coefficients: -0.45 and -0.32, respectively; linear regression ANCOVA).

## Discussion

In order to improve clinical outcomes following CLP, it is of high importance to evaluate the risk factors behind its failure in children and adolescents. This study highlighted the importance of considering clinical and radiological factors preoperatively, while the CP, the AP-TN coverage angles, and the GMFCS level seem to affect postoperative results. Also, when looking specifically at the CC joint, young age at the time of surgery and the preoperative CP angle were found to be the main risk factors for subluxation. Although an association was found between extensive LR and improved clinical results, when using univariate analysis, further investigations are necessary to confirm it.

This study failed to show a statistically significant relation between age and the overall clinical results according to Mosca’s criteria other than the one existing between young age at surgery and CC subluxation specifically, in opposition to Kadhim et al.'s findings. In a retrospective study of 78 patients (138 feet), they compared two treatment methods (CLP and subtalar fusion) and demonstrated that young age is a predicting factor of recurrence after surgery. However, in the aforementioned study, recurrence was defined by the need for additional intervention, with only 12 feet treated primarily with subtalar fusion having high GMFCS levels. This cannot be extrapolated to all the patients treated with CLP [13].

Based on Mosca’s clinical criteria, satisfactory results were more likely to occur in children with non-neurological cases (Table 2). When applied to cerebral palsy patients, CLP is more commonly performed in ambulant children with low GMFCS levels (I and II) and is intended to improve ambulation and reduce discomfort during walking. The majority of cerebral palsy children in this study were GMFCS I/II. Eight children with GMFCS III/IV, underwent the procedure for severe deformity interfering with walking and orthotic fitting (walking AFO or GRAFO). Clinical improvement was found to be inversely proportional to the GMFCS level with 71% (n=10/14) of non-satisfactory outcomes in children with GMFCS III/IV, and this was in accordance with Narang et al., where CLP was found safer in children with GMFCS I/II on a sample of 17 neurological feet with peroneus brevis lengthening [14]. Calcaneal lengthening alone may not be appropriate in patients with GMFCS III/IV, and additional surgeries such as medial column osteotomies, tendon transfers, and limited fusions may be required. This was confirmed by Cho et al. on a sample of 77 neurologic feet with a minimum follow-up of two years [15]. This is usually discovered intraoperatively when the abduction of the forefoot persists after calcaneal lengthening in severe and stiff deformities.

To our knowledge, this is the first study to evaluate the influence of extensive LR and different methods of bone fixation on predicting the final result of CLP. Owing to the difficulty in controlling the dorsal displacement of the calcaneal fragment with regards to the cuboid fragment, and in order to improve the stability of the osteotomy site and decrease the risk of rotatory subluxation as described by Siebert et al., it was decided to use two eight-plates instead of two pins [10]. Extensive LR was associated with a better satisfactory result than limited LR. This may be explained by our hypothesis of better 3D mobilization of the calcaneus during correction.

In this study, the use of eight-plates for osteotomy site fixation showed better results than K-wires, although not statistically significant. It is important to mention that the value of statistical significance as compared to clinical relevance is more and more questioned in many recent papers and forums, the last of which is published by Klouche et al., in which they concluded that the p-value is limited to 0.05 and may sometimes be of less clinical importance [16]. It would therefore be interesting to expand the study population in a prospective design and evaluate the results using these two fixation methods with the same predefined p-value (p<0.05).

Regardless of the patient's GMFCS level, age at surgery, etiology, preoperative severity of foot deformity, and surgical technical differences, CLP leads to an overall improvement in clinical and radiological parameters (as seen in Table 2 and Figure 6). This is in accordance with previous reports [17].

Based on this study’s findings, preoperative CP angle, AP-TN angle, and GMFCS level were the main factors influencing the overall clinical and radiological results. This is in concordance with Luo et al., who found, in 30 operated feet of patients with cerebral palsy, a significant relation between the CP and the AP-TN angles and the overall result [18]. Lengthening of the triceps muscle (gastrocnemius or Achilles) is necessary in quite a few cases in order to improve the CP angle. An increased graft size, a distal tibialis posterior tendon transfer, and a medial TN capsulorraphy may be necessary in some cases to improve the AP-TN angle. Those case-by-case additions to the original basic surgical technique were not evaluated in the current study for methodology reasons.

While it has been shown that CC joint subluxation is frequently encountered postoperatively [19], only 16% (n=13/80) of the evaluated feet in this study suffered from this complication. Among the 13 cases with this complication, seven (54%) had unsatisfactory results according to Mosca’s criteria. In addition, young age at the time of surgery and the preoperative CP angle were found to be the main risk factors for CC subluxation. These results may be related to the pain created by abnormal pressure across the CC joint or to suboptimal correction. Moreover, Ahn et al. reported a gradual improvement of the CC joint subluxation from 26% to 11% at the last follow-up, with a mean follow-up period of 25 months [20].

Subtalar arthroereisis has been lately used for the correction of planovalgus foot deformity. In a recent study on adult-acquired flatfeet, arthroereisis alone failed to correct all components of the deformity [21]. Bernasconi et al. reported their results of subtalar arthroereisis in children but did not find any significant impact of this technique on TN coverage [22]. Suh et al. reported better radiographic correction with CLP when compared to subtalar arthroereisis [23]. This remains in favor of CLP, which retains better correction in both AP and L-foot aspects.

Although the indications for surgery follow the reported international guidelines, the fact that they were set and the surgery executed by the same surgeon may be considered a confounding bias. Other limitations are the retrospective nature of the study, the heterogeneity of the sample, the relatively small patient population, and the short follow-up. A longer follow-up may reveal a greater incidence of deformity recurrence, as reported by Kadhim et al. [24]. In addition, because of the lack of availability of intermediate-term radiographic documents for some patients in this series, we decided to take into consideration only the last follow-up radiographs. The possibility of spontaneous improvement of CC subluxation with time, as reported in the literature, was therefore impossible to evaluate with accuracy. This study included all patients who failed conservative management for pain or discomfort during daily activities or school sports. None of the patients who had similar deformities but were asymptomatic were considered candidates for surgery. It is therefore impossible to identify a control group with similar symptoms that did not undergo surgery. Solutions to those limitations may be further studies on the subjects, preferably prospective, including a larger sample size, more involved surgeons, and independent examiners.

However, this is the first study evaluating the risk factors of CLP combining clinical, radiological, and surgical factors (limited LR, extensive LR, eight-plates, and K-wires) in a heterogeneous population containing not only neurologic but also idiopathic and dystrophic etiologies that constitute the majority of the planovalgus feet deformity cases. Moreover, a closer look was undertaken on the CC joint subluxation and its predictive factors as well as its relation to the final result.

## Conclusions

In conclusion, this study highlighted the risk factors for poor outcomes following the calcaneal lengthening procedure. It showed the importance of preoperative clinical and radiological evaluation. Better outcomes were associated with a low GMFCS level, a high calcaneal pitch, and a high TN coverage angle preoperatively. Surgical procedures performed at an early age or on a severe flatfoot deformity (low calcaneal pitch angle) were more associated with postoperative CC joint subluxation and poor results.
